# Ecology of Alpine Macrofungi - Combining Historical with Recent Data

**DOI:** 10.3389/fmicb.2017.02066

**Published:** 2017-10-26

**Authors:** Ivano Brunner, Beat Frey, Martin Hartmann, Stephan Zimmermann, Frank Graf, Laura M. Suz, Tuula Niskanen, Martin I. Bidartondo, Beatrice Senn-Irlet

**Affiliations:** ^1^Forest Soils and Biogeochemistry, Swiss Federal Institute for Forest, Snow and Landscape Research WSL, Birmensdorf, Switzerland; ^2^Community Ecology, WSL Institute for Snow and Avalanche Research SLF, Davos Dorf, Switzerland; ^3^Department of Comparative Plant and Fungal Biology, Royal Botanic Gardens, Kew, Richmond, United Kingdom; ^4^Department of Life Sciences, Imperial College London, London, United Kingdom; ^5^Biodiversity and Conservation Biology, Swiss Federal Institute for Forest, Snow and Landscape Research WSL, Birmensdorf, Switzerland

**Keywords:** *Dryas octopetala*, fungal communities, ectomycorrhiza, *Salix herbacea*, *Salix reticulata*, *Salix retusa*, *Salix serpyllifolia*, Swiss National Park

## Abstract

Historical datasets of living communities are important because they can be used to document creeping shifts in species compositions. Such a historical data set exists for alpine fungi. From 1941 to 1953, the Swiss geologist Jules Favre visited yearly the region of the Swiss National Park and recorded the occurring fruiting bodies of fungi >1 mm (so-called “macrofungi”) in the alpine zone. Favre can be regarded as one of the pioneers of alpine fungal ecology not least because he noted location, elevation, geology, and associated plants during his numerous excursions. However, some relevant information is only available in his unpublished field-book. Overall, Favre listed 204 fungal species in 26 sampling sites, with 46 species being previously unknown. The analysis of his data revealed that the macrofungi recorded belong to two major ecological groups, either they are symbiotrophs and live in ectomycorrhizal associations with alpine plant hosts, or they are saprotrophs and decompose plant litter and soil organic matter. The most frequent fungi were members of *Inocybe* and *Cortinarius*, which form ectomycorrhizas with *Dryas octopetala* or the dwarf alpine *Salix* species. The scope of the present study was to combine Favre's historical dataset with more recent data, either with the “SwissFungi” database or with data from major studies of the French and German Alps, and with the data from novel high-throughput DNA sequencing techniques of soils from the Swiss Alps. Results of the latter application revealed, that problems associated with these new techniques are manifold and species determination remains often unclear. At this point, the fungal taxa collected by Favre and deposited as exsiccata at the “Conservatoire et Jardin Botaniques de la Ville de Genève” could be used as a reference sequence dataset for alpine fungal studies. In conclusion, it can be postulated that new improved databases are urgently necessary for the near future, particularly, with regard to investigating fungal communities from alpine regions using new techniques.

## Introduction

The Swiss geologist Jules Favre was one of the first mycologists who explored the macrofungal communities in the alpine zones. Employed as a curator at the Natural History Museum of Geneva, he visited the region of the Swiss National Park (SNP) every summer between the years 1941 and 1957 making daily excursions to sample and record the fruiting bodies of fungi larger than 1 mm and easily visible to the naked eye (so-called “macrofungi”). He published the list of fungi occurring in the alpine zone (above timberline) in 1955 (Favre, 1955) and that one from the subalpine zone (below timberline) in 1960 (Favre, 1960). However, Favre (1955) not only presented a list of the occurring fungal taxa, moreover, he provided valuable ecological data, such as site location, elevation above sea level, geology, and associated plants. Hence, Jules Favre can be regarded not only as a pioneer of alpine mycology but also of alpine fungal ecology (Monthoux, 1973; Miskiewicz and Ronikier, 2002).

Other mycological studies from alpine zones were conducted decades later, mainly from the 1960s through the 1970s in the National Park “La Vanoise” in the French Alps (Bon and Géhu, 1973; Eynard, 1977; Kühner and Lamoure, 1986). In the arctic regions, the first mycofloristic investigations were made by Lange (1948, 1955, 1957) and Peterson (1977) in West Greenland, and by Ohenoja (1971) in Svalbard (Norway). In recent years, several studies of alpine fungi in association with the ectomycorrhizal host plants *Dryas octopetala, Salix* spp., *Kobresia myosuroides*, and *Bistorta vivipara* have been conducted in alpine regions of the Alps and Scandinavia. Investigations followed either a classical way by collecting fruiting bodies (Debaud et al., 1981; Senn-Irlet, 1987, 1988, 1993; Graf, 1994; Graf and Brunner, 1996) or a modern way by fingerprinting ectomycorrhizal root tips with genetic methods (Mühlmann and Peintner, 2008; Mühlmann et al., 2008; Ryberg et al., 2009, 2011; Bjorbækmo et al., 2010).

However, the vast mycological legacy of Favre (1955, 1960) still awaits a more in-depth analysis. Information from subalpine habitats (Favre, 1960) has been used in a few cases to analyse fungal communities of subalpine meadows and forests in the region of the SNP (Horak, 1985; Brunner and Horak, 1990). Because Favre (1955, 1960) also provided accurate information on sampling locations, his work can potentially serve as a basis for resurveys in the way historical records have been used for vascular plants (Pauli et al., 2012; Wipf et al., 2013). This task is urgent because biodiversity scenarios for the current century consistently forecast a loss of alpine habitats as well as of alpine plants (Thuiller et al., 2005; Engler et al., 2011), possibly resulting in a disappearance of their associated fungi. Few studies (Willis and MacDonald, 2011; Suz et al., 2015) highlight that the availability of long-term datasets is the strongest limiting factor in global change research, and that research linked to long-term monitoring plots will enhance the relevance of scientific results and conclusions to the practical application of mitigation measures.

In the present study, we aimed to analyse the comprehensive myco-ecological study of Favre (1955). The main goal was to locate the most relevant sampling sites of the fungi in the alpine zones of the SNP, to identify the fungal lifestyles, and to characterize the fungal communities associated to *Dryas octopetala* and the alpine dwarf willows *Salix* spp. In addition, we aimed to combine the database of Favre (1955) with the “SwissFungi” inventory (Senn-Irlet, 2010) as well as with more recent literature from the Swiss, French and German Alps in order to provide a more comprehensive basis on the diversity of alpine macrofungi. Furthermore, we intended to compare Favre's data with the newest results from high-throughput DNA sequencing (HTS) analyses from soils of the Swiss Alps (e.g., Frey et al., 2016). With the comparison of the fungal lists it was of interest, whether and which taxa listed by Favre (1955) could also be found with these new HTS techniques. Our ultimate goal was to establish a firm foundation with respect to future challenges related to alpine fungal ecology.

## Materials and methods

### The historical dataset of Favre (1955)

The main source of information was the book of Favre (1955). Because he listed the fungi taxonomically in his book including detailed ecological information assigned to each fungal species, the locations, the elevation above sea level, the geology, and the associated plants had to be extracted in a meticulous way. Additional information was found at the Botanical Garden of Geneva (“Conservatoire et Jardin Botaniques de la Ville de Genève”) where the field-book of Jules Favre is kept. In that field-book, each one-day excursion from the years 1942 to 1957 is listed with the date, the routes, and locations in the alpine zone underlined. The Botanical Garden of Geneva also preserves Favre's dried fungal material (“exsiccata”) including all the relevant information. The exsiccata are listed and displayed publicly (http://www.ville-ge.ch/musinfo/bd/cjb/chg/result.php?type_search=simple&lang=fr&criteria=favre&mode=tout).

### The Swiss National Park

The SNP (http://www.nationalpark.ch) was founded in 1914 and, is the oldest National Park of the Alps. It is located in the southeast of Switzerland and has 170 km^2^ of natural landscape including subalpine forests, alpine grassland, and nival peaks up to 3,174 m of height. The SNP is situated on a mosaic of various bedrocks, but dominated by dolomitic limestone and only occasionally interrupted by various other limestone formations. Some parts consist of acidic bedrock formed by granite gneiss of crystalline base or of sandstones, shales, and psephites of the Permian (“Münstertaler Verrucano”). What Favre (1955) called “triassic limestone” corresponds to a quaternary moraine, to dolomite, or to coral limestone. “Gneissic” and “granite soils” correspond to a muscovite granite gneiss and “verrucano” corresponds to a conglomerate of cemented gravel, sand, and rounded stones of different sizes. The predominant geological formations of the sampling area can be viewed with the Swiss geology data viewer (https://map.geo.admin.ch).

### Naming the fungal taxa and assigning their lifestyles

Naming of the fungal species followed the “Index Fungorum” (http://www.indexfungorum.org/names/names.asp). According to that “Index,” many genus names have changed since Favre's descriptions, e.g., *Rhodophyllus* changed to *Entoloma, Geophila* to *Psilocybe*, and the genus *Hygrophorus* has been splitt into several genera, e.g., *Hygrophorus s.s., Hygrocybe, Gliophorus*, or *Cuphophyllus* (Lodge et al., 2014). However, species of *Hygrophorus s.s*. were not found by Favre (1955) since they form ectomycorrhiza (ECM) with trees below the alpine zone.

The lifestyles of fungi were assigned according to the fungal guilds published in Nguyen et al. (2016), e.g., saprotrophs, ectomycorrhizal fungi, lichenized fungi, and many others. Due to new scientific evidence, members of the genus *Helvella* are classified as ectomycorrhizal fungi (Rinaldi et al., 2008; Tedersoo et al., 2010), and the genera *Hygrocybe* and *Entoloma* are considered saprotrophs (Högberg et al., 1999; Hobbie et al., 2001), although there are indications that members of the latter two genera can symbiotically associate with roots (e.g., Tello et al., 2014).

### Recent data from the Swiss, French, and German Alps

In order to compare the historical records of Favre (1955) with more recent fungal records collected in the Alps, the “SwissFungi” database (http://www.swissfungi.ch) and some major studies from the Swiss (Senn-Irlet, 1987; Graf, 1994), French (Bon and Géhu, 1973; Kühner and Lamoure, 1986), and German Alps (Schmid-Heckel, 1985) were considered. The “SwissFungi” database is part of the Swiss national data center for biodiversity initiated by the Federal Office for the Environment (FOEN) and located at the Swiss Federal Institute for Forest, Snow and Landscape Research WSL in Birmensdorf (Switzerland, Senn-Irlet, 2010). The main objective of “SwissFungi” is to present updated maps of the distribution of fruiting bodies for each species of fungi in Switzerland. These distribution maps serve as a base for the elaboration of the Red List of threatened species and inform authorities about the presence of species requiring protection in each region. Since 1992, more than half a million records of fungi have been stored, with a focus on macrofungi, in order to deepen the ecological knowledge about the species diversity at a national level.

### High-throughput DNA sequencing techniques of Swiss alpine soils

In order to compare the fungal records of Favre (1955) with most recent fungal records from the Alps, the lists of newly generated data using novel high-throughput DNA sequencing (HTS) techniques of alpine soils in Switzerland were used. Such novel DNA sequencing techniques allow the analysis of the entire fungal community in soils in different ecosystems, including alpine soils (Hartmann et al., 2014, 2015; Pellissier et al., 2014). HTS techniques allow an in-depth description of the microbial diversity and their potential relative abundance by massively parallel DNA sequencing to increase the number of sampled PCR amplicons in environmental surveys (Sogin et al., 2006). Here we compare the fungal records of Favre (1955) with the HTS results from the fungal communities of two studies carried out with soils of the Swiss Alps. Rime et al. (2015) investigated soils of a recently deglaciated area in front of the “Damma” glacier (2,000–2,100 m a.s.l.), and Frey et al. (2016) investigated soils near the summit of “Muot da Barba Peider” (2,960 m a.s.l.) with permanently frozen soils (“permafrost”) in deeper layers. In both studies, the internal transcribed spacer region 2 (ITS2) of the eukaryotic ribosomal operon (fungi and some groups of protists and green algae) was amplified (Frey et al., 2016).

## Results

### Excursion destinations of Favre (1955)

**Figure 1:**
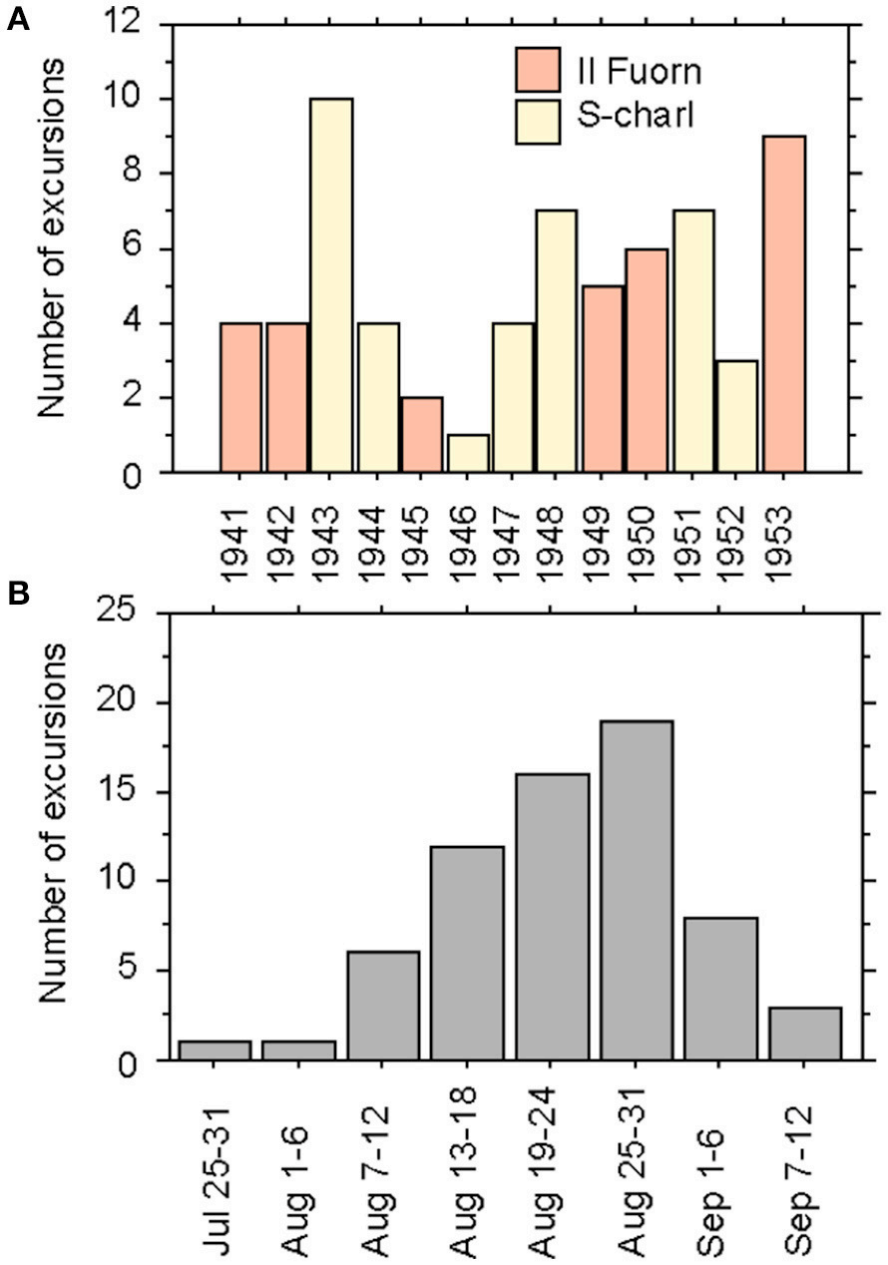
Bar charts of the excursions made by Favre (1955) in the region of the Swiss National Park according to his field-book. **(A)** Number of the excursions made per year from 1941 to 1953 in the surroundings of Il Fuorn (orange) and S-charl (yellow), and **(B)** number of excursions made per time period in the months July to September.

Between 1941 and 1957, Favre usually spent several weeks during the summer months at one location and made daily excursions into the subalpine and alpine zones. Because of his advanced age, he stopped the excursions into the alpine zones in 1953. In total, he made 66 excursions, with one to ten excursions per year. He started his excursions either from Il Fuorn and Pass dal Fuorn (during six years) or from the village of S-charl (during 7 years) (Figure 1A). His earliest excursion started in late July and his latest in early September, but with a clear majority of the excursions taking place in the second half of August (Figure 1B).

**Figure 2:**
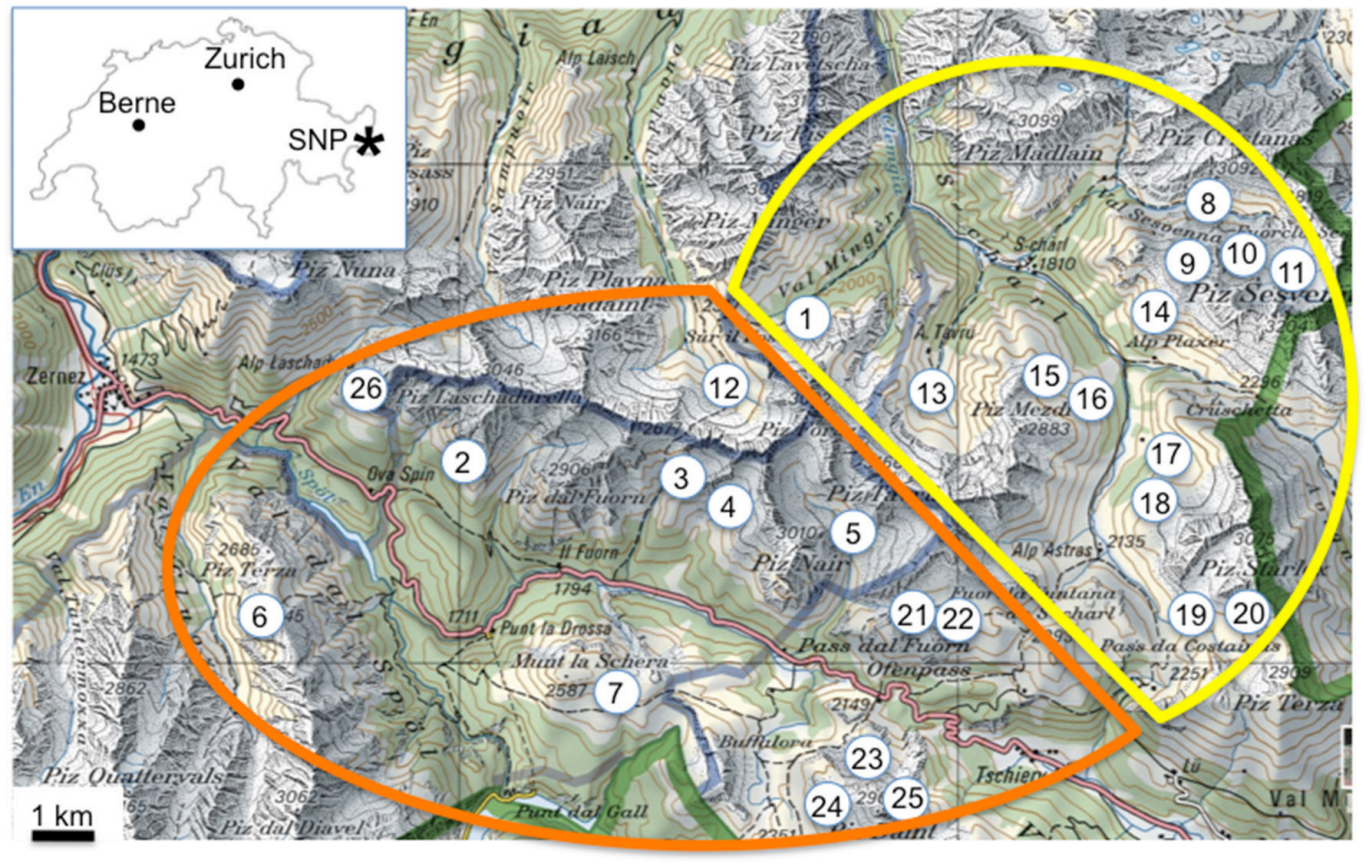
Overview of the sampling sites of Favre (1955) in the region of the Swiss National Park (SNP). The sampling sites are in the surroundings of Il Fuorn (orange line) and of S-charl (yellow line). The inset graph shows the location of the SNP (^*^) within Switzerland. The green solid line shows the border between Switzerland and Italy, and the blue solid line shows the border of the SNP. Reproduced with permission from swisstopo (JA100118). Sites within SNP: (1) Val Mingèr, (2) Murteras da Grimmels, (3) Val dal Botsch, (4) Val Stabelchod, (5) Val Nüglia, (6) Alp Murtèr, Piz Murtèr, (7) Munt la Schera, Munt Chavagl. Sites outside of SNP: (8) Marangun, (9) Blaisch dals Manaders, (10) Ils Lajets (11) source of Sesvenna, (12) Val Plavna, (13) Blaisch Bella, Val Tavrü, (14) Munt Plazèr, (15) Mot dal Gajer, (16) Piz Mezdi, (17) Valbella, (18) Murters da Tamangur, (19) Costainas, (20) Piz Starlex, (21) Chaschlot, (22) Munt da la Bescha, (23) Murtaröl, (24) Taunter Pizza, Jufplaun, (25) Piz Daint, (26) Val Laschadura, Foppinas.

From S-charl most of the excursions lead Favre into the nearby valleys Val S-charl (16x) and Val Sesvenna (13x) (Table 1). In the surroundings of Il Fuorn and Pass dal Fuorn he mainly visited the areas of Murtaröl (8x) and Munt la Schera (5x), as well as the nearby valleys Val dal Botsch (5x) and Val Nüglia (3x) (Table 1). An overview of the SNP and its surroundings, including the two main excursion areas Il Fuorn and S-charl, is given in Figure 2.

**Table 1:** Excursion destinations, regions, and number of excursions (n) according to the field-book of Favre (1955).

**Excursion destination**	**Region**	***n***
**IL FUORN AND SURROUNDING AREAS**		
Murtaröl, Piz Daint, Taunter Pizza, Fuorn	Pass dal Fuorn	8
Val Nüglia	Pass dal Fuorn	3
Munt da la Bescha	Pass dal Fuorn	3
Val dal Botsch, Margunet, Val Stabelchod	Il Fuorn	5
Munt la Schera, Munt Chavagl	Il Fuorn	5
Murteras da Grimmels, Murteras d'Ivraina, Val Laschadura, Foppinas	Il Fuorn	3
Piz Murtèr	Il Fuorn	2
Val Mingèr	Val Mingèr	1
Total		30
**S-CHARL AND SURROUNDING AREAS**		
Ils Lajets	Val Sesvenna	4
Source of Sesvenna	Val Sesvenna	3
Blaisch dals Manaders	Val Sesvenna	3
Marangun	Val Sesvenna	2
Mot da l'Hom	Val Sesvenna	1
Mot dal Gajer	Val S-charl	4
Murters da Tamangur	Val S-charl	3
Costainas	Val S-charl	3
Valbella	Val S-charl	2
Munt Plazèr	Val S-charl	2
Mot Mezdi, Mot Madlain	Val S-charl	2
Blaisch Bella	Val Tavrü	2
Val Mingèr	Val Mingèr	5
Total		36

### Sampling sites of Favre (1955)

The analysis of Favre's (1955) book and his field-notes revealed about 26 sites where he sampled fungal fruiting bodies (Table 2, Figure 2). The majority of the sampling sites were on calcareous rocks (15 of 26 sites; dolomite, quaternary moraine, coral limestone) where the calciphilic ectomycorrhizal plants *Dryas octopetala, Salix retusa, S. reticulata*, and *S. serpyllifolia* grow. Ten sites on muscovite granite gneiss and one on verrucano were preferentially colonized by the also ectomycorrhizal, but acidophilic, *Salix herbacea*. A few sites on the quaternary moraine, dolomite, and muscovite granite gneiss hosted *D. octopetala* as well as *S. herbacea* (Table 2).

**Table 2:** Sampling sites and number of fungal species of Favre (1955).

**Sampling sites**	**Coordinates (N/W)**	**Elevation (m a.s.l)**	**Geology**	**Fungal records in association**			**No**.
				***D***	***Srrs***	***Sh***	
Murtaröl	46°38′/10°17	2,250–2,500	Quaternary moraine	24	11	-	23
Piz Mezdi	46°41′/10°20′	2,200–2,500	Quaternary moraine	16	5	-	16
Val Mingèr	46°43′/10°16′	1,900–2,550	Quaternary moraine	14	8	-	1
Valbella	46°41′/10°22′	2,150–2,200	Quaternary moraine	9	13	-	17
Alp Murtèr/Piz Murtèr	46°41′/10°09′	2,300–2,700	Coral limestone	6	9	-	6
Val Stabelchod	46°41′/10°16′	2,250–2,600	Quaternary moraine	6	8	-	4
Chaschlot	46°39′/10°17′	2,250–2,500	Dolomite	8	1	-	21
Murteras da Grimmels	46°41′/10°11′	2,400–2,650	Dolomite	6	1	-	2
Piz Daint	46°38′/10°17′	2,300–2,450	Dolomite	3	2	-	25
Val Plavna/Spadla Sura	46°42′/10°16′	2,100–2,400	Quaternary moraine	2	2	-	12
Blaisch Bella/Val Tavrü	46°41′/10°18′	2,300–2,450	Muscovite granite gneiss	1	7	-	13
Val dal Botsch	46°41′/10°15′	2,000–2,800	Quaternary moraine	20	8	3	3
Val Nüglia	46°41′/10°17′	2,000–2,600	Quaternary moraine	18	5	3	5
Munt la Schera/M. Chavagl	46°39′/10°13′	2,000–2,550	Dolomite	15	7	2	7
Munt da la Bescha	46°39′/10°18′	2,200–2,550	Dolomite	6	3	4	22
Blaisch dals Manaders	46°43′/10°22′	2,400–2,700	Muscovite granite gneiss	3	1	5	9
Costainas	46°39′/10°23′	2,400–2,650	Muscovite granite gneiss	2	5	2	19
Marangun	46°44′/10°23′	2,200–2,400	Muscovite granite gneiss	2	1	3	8
Murters da Tamangur	46°40′/10°22′	2,350–2,500	Muscovite granite gneiss	1	1	3	18
Mot dal Gajer	46°42′/10°20′	2,350–2,750	Muscovite granite gneiss	1	1	1	15
Val Laschadura/Foppinas	46°42′/10°09	2,100–2,700	Muscovite granite gneiss	1	1	1	26
Ils Lajets	46°43′/10°23′	2,400–2,700	Muscovite granite gneiss	-	1	17	10
Source of Sesvenna/moraine	46°43′/10°24′	2,250–2,650	Muscovite granite gneiss	-	3	5	11
Munt Plazèr	46°42′/10°23′	2,600–2,650	Muscovite granite gneiss	-	3	5	14
Taunter Pizza/Jufplaun	46°37′/10°17′	2,300–2,650	Verrucano	-	5	5	24
Piz Starlex	46°39′/10°24′	2,250	Dolomite	2	-	-	20

*The macrofungi were growing in association with the calciphilic host plants Dryas octopetala (D) and Salix reticulata, S. retusa and S. serpyllifolia (Srrs), and with the acidophilic host plant Salix herbacea (Sh). The sampling sites are listed with their coordinates, elevation and the predominant geological bedrock*.

*Number of fungal records, 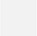
1–10, 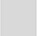
11–20, 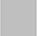
21–30, 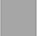
31–40. No., Site number according to Figure 2*.

Some sites have a large elevation span, e.g., from about 2,000 to 2,600 m a.s.l. for Val Nüglia and Munt la Schera, and other sites have only a narrow range, e.g., from 2,150 to 2,200 m a.s.l. for Valbella and from 2,600 to 2,650 m a.s.l. for Munt Plazèr (Table 2).

The most relevant sampling sites with >30 fungal records are the sites Murtaröl and Val dal Botsch, and with 21–30 fungal records the sites Piz Mezdi, Val Mingèr, Valbella, Val Nüglia, and Munt la Schera/Munt Chavagl (Table 2. dark gray shadows). The largest number of fungal species in association with *D. octopetala* was observed by Favre (1955) in the calcareous sites Murtaröl (24 species), Val dal Botsch (20), and Val Nüglia (18), in association with the calciphilic *Salix* species in Valbella (13) and Murtaröl (11), and association with the acidophilic *S. herbacea* in Ils Lajets (17) (Table 2).

### Fungal taxa recorded by Favre (1955)

In his book, Favre (1955) listed in total 202 fungal species in 1302 records mainly belonging to macrofungi. The majority of recorded species belonged to the Basidiomycota, whereas he only recorded 12 species belonging to the Ascomycota. A large majority of the fungi were already known from other regions of Europe. At least 63 of them were known from forests or meadows, seven from dung, and 11 from mires and swamps (Favre, 1955). However, Favre (1955) designated about 121 species as mainly alpine species. About one-third of them were unknown to him, and, thus, he described them as new species. In total, he described 46 new species and classified the majority of them as members of the genera *Inocybe* and *Cortinarius*. Additionally, for 20 species that were already known, he described several variations and forms. In some cases, other mycologists assigned such forms and variations to new species at a later stage, e.g., *Hebeloma alpinum* (= *Hebeloma crustuliniforme var. alpinum* according to Favre, 1955) by G. Bruchet, *Inocybe taxocystis* (= *I. decipientoides var. taxocystis* according to Favre, 1955) by E. Horak, or *Amanita oreina* (= *A. vaginata f. oreina* according to Favre, 1955) by R. Heim. On the other hand, two taxa described by Favre (1955), *Marasmius obscurus* and *Rhodophyllus umbella*, have not been accepted because no deposited exsiccata are available. All these modifications resulted in an overall species number of 204 instead of 202 as listed in Favre (1955) (see also below).

**Figure 3:**
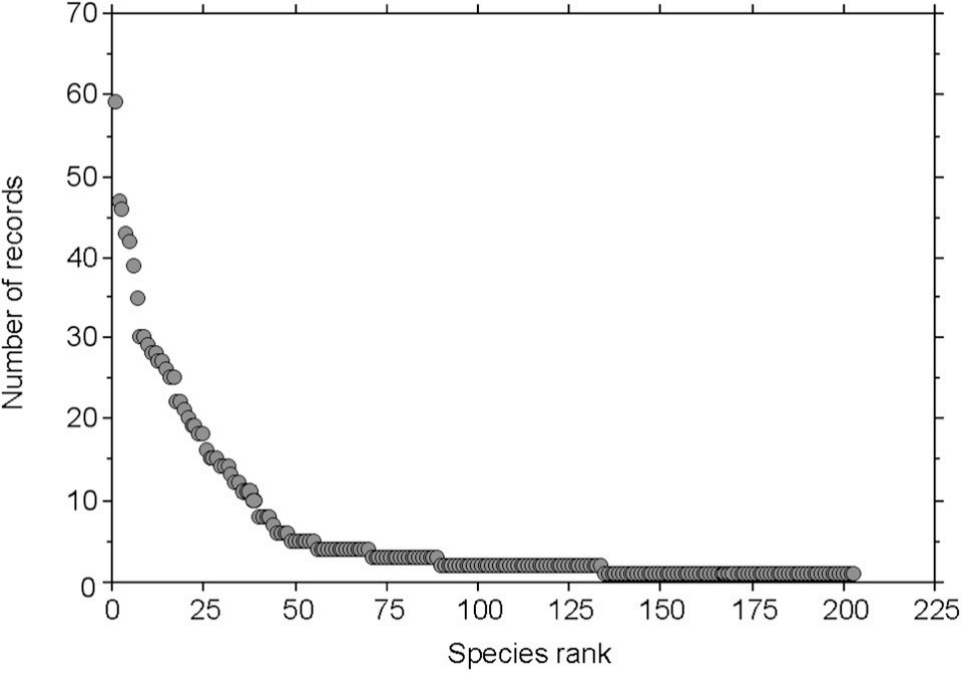
Rank-abundance diagram of fungal taxa recorded by Favre (1955). The rank-abundance diagram displays the contribution of the fungal taxa (number of records) to a community against their ranks, with the most common species ranked first and the most rare species ranked last.

The genera of fungi with the highest number of recorded species were *Inocybe* with 40 species, *Cortinarius* with 26 species, and *Entoloma* with 14 species. Other abundant fungi were members of the genera *Hygrocybe, Omphalina, Marasmius*, and *Psilocybe*. The individual species with the largest number of observations by Favre (1955) were *Inocybe rimosa, I. dulcamara, Hebeloma marginatulum*, and *Hygrocybe conica*, each of which had >40 records (Tables 3, 4). A rank-abundance diagram shows that most of the fungal taxa were found only once or only a few times: he recorded about 34% of the fungi only 1 time, and 76% of the fungi ≤5 times (Figure 3). The highest elevation from where Favre recorded fungal fruiting bodies was at 2,850 m a.s.l. (*Omphalina pyxidata, Arrhenia griseopallida*.)

**Table 3:** Abundant ectomycorrhizal fungal species (>5 records) of Favre (1955).

**Species name**	**Species name (in Favre, 1955)**	**Fungal records in association with**		
		***Dryas o***.	***Salix rrs***.	***Salix h***.
**ASSOCIATED WITH ALL *DRYAS* AND *SALIX* SPECIES**				
*Inocybe rimosa*	(= *I. fastigiata, f. alpestris+alpina*)		59	
*Inocybe dulcamara*	*(= I. dulcamara, all forms)*		47	
*Hebeloma marginatulum* ^*^	(= *H. versipelle var. marginatulum*)		46	
*Laccaria laccata*	(= *L. laccata var. montana*)		43	
Hebeloma alpinum^*^	(= *H. crustuliniforme var. alpinum*)		35	
Inocybe geraniodora^*^			29	
Cortinarius minutulus^*^			27	
Cortinarius tenebricus^*^			27	
*Hebeloma mesophaeum*			20	
*Cortinarius hinnuleus*	(= *C. hinnuleus, all forms and varieties)*		16	
*Cortinarius anomalus*			15	
*Inocybe nitidiuscula*	*(= I. Friesii, all forms;*		15	
	*= I. ovalispora-subbrunnea f. brunneola)*			
*Laccaria pumila*	(= *L. laccata var. pumila*)		12	
Cortinarius pauperculus^*^			8	
*Inocybe decipiens*	*(= I. decipiens, all varieties)*		8	
*Helvella corium*	*(= H. arctica var. macrosperma; = H. corium f. alpestris)*		6	
**ASSOCIATED WITH CALCIPHILIC *DRYAS* AND *SALIX* SPECIES**				
Inocybe canescens^*^			25	-
*Inocybe fuscomarginata*			14	-
Inocybe oreina^*^			6	-
**ASSOCIATED WITH ALL *SALIX* SPECIES**				
*Cortinarius alpinus*		-	26	
*Cortinarius cinnamomeus*		-	22	
Cortinarius phaeopygmaeus^*^		-	14	
*Inocybe praetervisa*		-	14	
Amanita oreina^*^	(= *A. vaginata f. oreina*)	-	11	
**ASSOCIATED WITH *SALIX HERBACEA***				
*Russula pascua*	*(= R. xerampelina var. pascua)*	-	-	21
Lactarius nanus^*^		-	-	18
*Inocybe lacera*	*(= I. lacera, all forms and varieties; = I. rhacodes)*	1	-	14

*The macrofungi were growing in association with the calciphilic Dryas octopetala (Dryas o.) and Salix reticulata, S. retusa and S. serpyllifolia (Salix rrs.) on calcareous rocks, and with the acidophilic Salix herbacea (Salix h.) on gneiss or verrucano*.

*Number of fungal records, 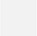
6–10, 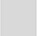
11–20, 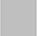
21–30, 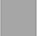
31–40, 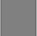
41–60*.

* *Named by Favre*.

**Table 4:** Abundant saprotrophic fungal species (>5 records) of Favre (1955).

**Species name**	**Species name (in Favre, 1955)**	**Fungal records in association with**					
		***D***	***Srrs***	***Sh***	**Gc**	**Ga**	**B**
**GROWING UBIQUITOUS**							
*Hygrocybe conica*	*(= Hygrophorus conicus et var. nigresc.)*			42			-
*Psilocybe montana*	*(= Geophila atrorufa)*			30			-
**GROWING WITH** ***DRYAS OCTOPETALA***							
Clitocybe dryadicola^*^	(= *C. rivulosa var. dryadicola*)	39	-	-	-	-	-
*Rhizomarasmius epidryas*	(= *Marasmius epidryas*)	30	-	-	-	-	-
Infundibulicybe lateritia^*^	(= *Clitocye lateritia*)	28	-	-	-	-	-
*Gymnopus dryophilus*	(= *Marasmius dryophilus*)	11	-	-	-	-	-
**PREFERENTIALLY GROWING WITH ALL** ***SALIX*** **SPECIES**							
Arrhenia obatra^*^	*Omphalina obatra*	-		19	-	-	-
*Entoloma conferendum*	*(= Rhodoph. staurosporus, var. Rickeni)*	-		5	-	2	-
**PREFERENTIALLY GROWING WITH** ***SALIX HERBACEA***							
Entoloma alpicola^*^	*(= Rhodophyllus clypeatus f. alpicolus;* = *Rhodophyllus sericeus var. nanus)*	-	-	11	-	11	-
**PREFERENTIALLY GROWING IN GRASSLANDS**							
*Bovista nigrescens*		1	-	-		27	-
*Lycoperdon umbrinum*		1	1	-		23	-
*Bovista tomentosa*		3	1	-		14	-
*Cuphophyllus pratensis*	*(= Hygrophorus pratensis)*	-	-	-		12	-
*Lycoperdon nigrescens*		1	1	-		11	-
*Bovista plumbea*		1	-	-		10	-
*Melanoleuca strictipes*	*(= M. evenosa)*	-	-	-		8	-
*Entoloma sericeum*	*(= Rhodophyllus sericeus f flexipes + f. rubellotactus + f. luridofuscus)*	1	-	1		6	2
*Hygrocybe marchii*	*(= Hygrophorus Marchii)*	3	-	-		5	-
*Melanoleuca stridula*		3	-	-		3	-
**GROWING IN BOGS**							
Galerina annulata^*^	*(= Galera rubiginosa var. annulata)*	-	-	-	-	-	6

*The macrofungi were growing in association with calciphilic Dryas octopetala (D) and/or Salix reticulata, S. retusa and S. serpyllifolia (Srrs) and/or acidophilic Salix herbacea (Sh), and/or in alpine grassland on calcareous (Gc) and/or acidic soils (Ga) and/or in bogs (B)*.

*Number of fungal records, 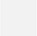
6-10, 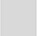
11-20, 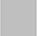
21-30, 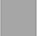
31-40, 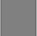
41-60*.

* *Named by Favre*.

The majority of the fungi recorded by Favre (1955) are symbiotrophs or saprotrophs. Among the symbiotrophs, ectomycorrhizal fungi (ECM) are dominant, and only a few species were lichenized, such as the basidiolichen *Lichenomphalina* spp. Other lichenized fungi were not recorded, although known to be abundant in the alpine zone. Overall, 95 ectomycorrhizal, 105 saprotrophic, and 4 lichenised taxa were recorded, resulting in a total of 204 taxa (Tables 3, 5, Tables S1, S2).

**Table 5:** Number of fungal records of Favre (1955) not growing in association with *Dryas* or *Salix*.

**Species name**	**Species name (in Favre, 1955)**	**Host or substrate**	**No. of records**
**ECTOMYCORRHIZAL SPECIES**			
*Cantharellus cibarius*		*Juniperus communis*	1
*Inocybe leptophylla*	*(= I. Casimiri)*	Peat bog	1
*Russula sanguinea*		*Juniperus communis*	2
*Thelephora terrestris*	*(= Phylacteria terrestris)*	Black humic soil	1
*Tricholoma inamoenum*		*Juniperus communis*	1
**LICHENIZED SPECIES**			
*Lichenomphalia alpina*	*(= Omphalina flava)*	On acidic soils	1
*Lichenomphalia hudsoniana*	*(= Omphalina luteolilacina)*	On acidic soils	2
*Lichenomphalia umbellifera*	*(= Omphalina umbellifera)*	On acidic soils	19
*Lichenomphalia velutina*	*(= Omphalina grisella)*	On acidic soils	2
**COPROPHILOUS SPECIES**			
*Stropharia umbonatescens*	*(= Geophila umbonatescens)*	Cow dung (“cowpat”)	1
*Conocybe coprophila*		Cow dung (“cowpat”)	1
*Panaeolus papilionaceus*	*(= P. campanulatus)*	Cow dung (“cowpat”)	several
*Panaeolus semiovatus*	*(= P. separatus)*	Cow dung (“cowpat”)	3
*Coprinus niveus*		Cow dung (“cowpat”)	2

*The macrofungi were either ectomycorrhizal, lichenized, or coprophilous*.

### Ectomycorrhizal fungi recorded by Favre (1955)

Overall, Favre (1955) recorded 27 ECM fungal taxa with >5 records (Table 3). Among them are *Inocybe rimosa s.l., I. dulcamara s.l., Hebeloma marginatulum, H. alpinum* and *Laccaria laccata*. However, only *Hebeloma marginatulum* and *H. alpinum* seem to be confined to alpine habitats (Table 3), whereas the *Inocybe* species and *L. laccata* are known from lower altitudes as well. Most of the ECM taxa, however, were only occasionally found (≤5 records), and some were recorded only once.

**Figure 4:**
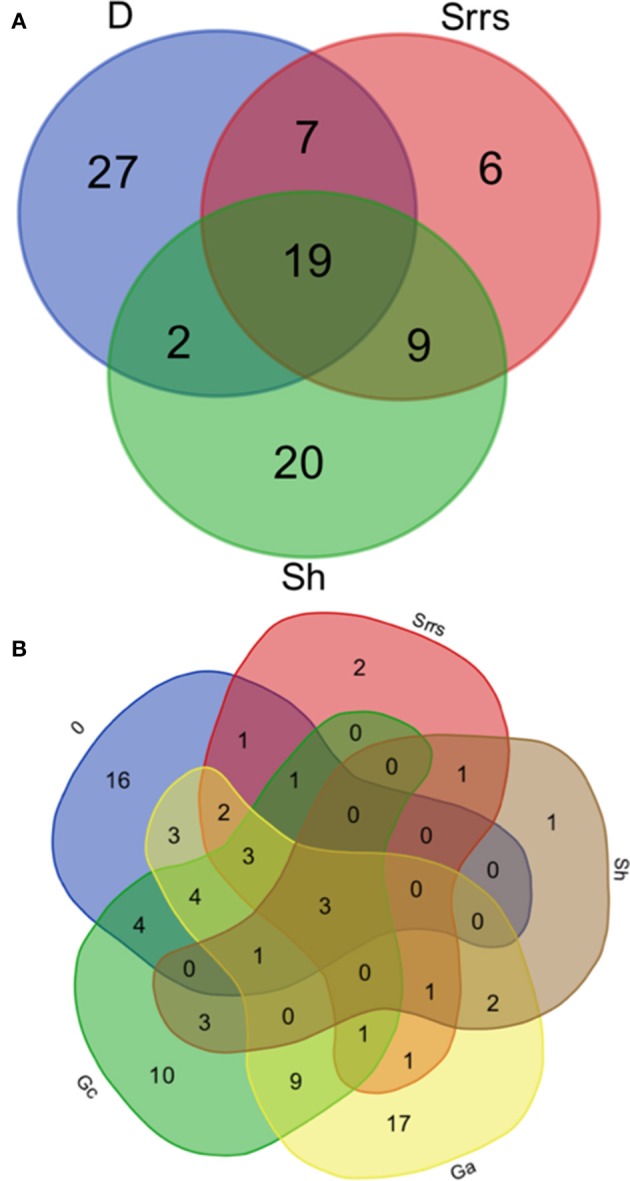
Venn diagrams of the fungal species recorded by Favre (1955). **(A)** Venn diagram displaying the relationships between ectomycorrhizal fungal species growing in association with *Dryas* (D) on calcareous bedrock, with alpine *Salix* (Srrs) on calcareous bedrock, and with alpine *Salix* (Sh) on acidic bedrock. **(B)** Venn diagram displaying the relationships between the saprotrophic fungal species growing in association with *Dryas* (D) on calcareous bedrock, with alpine *Salix* (Srrs) on calcareous bedrock, with alpine *Salix* (Sh) on acidic bedrock, with alpine grassland on acidic bedrock (Ga), and with alpine grassland on calcareous bedrock (Gc). D*, Dryas octopetala;* Srrs*, Salix reticulata, S. retusa, S. serpyllifolia;* Sh*, Salix herbacea;* Ga, grassland on acidic bedrock; Gc, grassland on calcareous bedrock.

From the whole range of the ECM species found by Favre (1955) 22% were found to be associated with *Dryas* as well as with all of the four dwarf willows (Table 3, Figure 4A). Thirty percent of the ECM taxa grew specifically with *Dryas*, 22% specifically with *Salix herbacea*, and 7% specifically with the three *Salix* species on calcareous soils (Table S1). About one third of these ECM fungal taxa were newly described by Favre (1955) (indicated with an ^*^ in the lists).

In most cases Favre (1955) indicated the putatively associated alpine plants *Dryas octopetala* and the four dwarf willows. Only in a few cases, Favre cited other potential hosts, such as *Juniperus communis* (presumed mycorrhizal partners: *Cantharellus cibarius, Russula sanguinea, Tricholoma inamoenum*; Table 5), *Loiseleuria procumbens* (*Cortinarius cinnamomeus, Hebeloma marginatulum, Russula emetica, R. pascua*), and *Bistorta vivipara* (*Inocybe dulcamara*). However, only *B. vivipara* is known to host ECM fungi, whereas *J. communis* hosts preferentially arbuscular mycorrhizal (AM) fungi and *L. procumbens* ericoid fungi (Read and Haselwandter, 1981). Besides *D. octopetala, Salix* spp. and *B. vivipara*, it is known that *Kobresia myosuroides, Arctostaphylos uva-ursi, A. alpina*, and *Helianthemum* spp. also form ECM, and even some *Carex* species may live in an ectomycorrhizal symbiosis (see also Read and Haselwandter, 1981).

### Saprotrophic fungi recorded by Favre (1955)

About 20 taxa of the more than 100 saprotrophic fungi were abundantly found with >5 records. The most common species were *Hygrocybe conica* and *Psilocybe montana*, which were recorded in grasslands as well as in mats of *Dryas* and all four dwarf willows (Table 4). This clearly dominant group of fungi represented by 36 species grew exclusively in grasslands: 17 taxa on acidic soils, 10 taxa on calcareous soils, and 9 taxa on both soil types (Table 4, Figure 4B, Table S2). Another large group with 16 taxa was found exclusively in association with *Dryas*, e.g., *Clitocybe dryadicola, Rhizomarasmius epidryas, Infundibulicybe lateritia*, and *Gymnopus dryophilus*, probably decaying leaves or woody parts of *Dryas*. In contrast, *Arrhenia obatra* grew mainly in association with *Salix spp*. on calcareous as well as on acidic soil. Some abundant taxa found in the alpine meadows are also known from meadows of lower altitudes, e.g., *Bovista nigrescens* and *Lycoperdon umbrinum* (Table 4).

Fungi recorded in bogs and swamps were mainly confined to these types of habitat. In total, 14 taxa were recorded, e.g., *Galerina annulata, Bovistella paludosa* and *Psilocybe elongata* (Table 4, Table S2). Five taxa were found to grow exclusively in “cowpads” on alpine pastures outside the SNP, e.g., *Panaeolus semiovatus* (Table 5).

### Exsiccata from Favre (1955) deposited at the conservatory and botanical garden in Geneva

A large proportion of the fungal specimens recorded including those newly named in Favre (1955), are stored at the “Conservatoire et Jardin Botaniques de la Ville de Genève.” The list of fungi is provided at http://www.ville-ge.ch/musinfo/bd/cjb/chg. Overall, 392 exsiccata of fungal specimens collected by J. Favre are deposited in Geneva, of which 249 specimens were from the region of the SNP (Favre, 1955, 1960). Of these, 188 were sampled in the alpine zone above 2,000 m a.s.l., with six specimens classified as “typus,” 20 as “holotypus,” four as “isotypus,” 28 as “lectotypus,” 124 as “syntypus,” three as “isosyntypus,” two as “isolectotypus,” and one was not assigned to a particular type. In our study, and according to the “Index Fungorum,” we recorded 59 type specimens indicated with (^*^) (Tables 3, 4, Tables S1, S2). The majority are members of the genera *Inocybe* (20 newly described taxa) and *Cortinarius* (20), followed by *Entoloma* (3), and *Hebeloma, Arrhenia*, and *Clitocybe* (each 2). With so many type specimens assigned, all sampling sites of Favre (1955) (see Table 2) within and around the SNP can be considered as “type locations.”

In recent years, several microscopical re-examinations of Favre's dried specimens from the botanical garden in Geneva have led to taxonomic revisions of whole genera, subgroups, or single specimens, e.g., Horak (1987a) and Senn-Irlet (1992) of *Cortinarius*, Horak (1987b) of *Inocybe*, Horak (1993) of *Entoloma*, and (Beker et al., 2016) of *Hebeloma*. However, DNA analyses of Favre's type specimens are rare and have only been used in a few studies. Altogether, the DNA sequences of eight type specimens (e.g., *Cortinarius, Entoloma, Hebeloma*, and *Inocybe* species) have been included recently in mycological surveys and phylogenetic analyses by Cripps et al. (2010), Kokkonen and Vauras (2012), Kokkonen (2015), Eberhardt et al. (2015), Liimatainen et al. (2015), and Garnica et al. (2016) (Table 6).

**Table 6:** ITS sequences of type specimens of Favre (1955) deposited at the NCBI GenBank.

**Species name**	**Species name (in Favre, 1955)**	**NCBI accession no**.
*Entoloma anthracinum*	*(=Rhodophyllus anthracinus)*	LN850598
*Hebeloma alpinum*	*(=H. crustuliniforme var. alpinum)*	KM390768
*Inocybe dulcamara*	*(=I. dulcamara f. pygmaea)*	GU980629
*Inocybe giacomi*		JN580864
*Inocybe taxocystis*	*(=I. decipientoides var. taxocystis)*	JN580884

### The “SwissFungi” database and other records from the Swiss, French, and German Alps

A check through the “SwissFungi” database in connection with other studies from the alpine zone from the Swiss, French, and German Alps revealed that the diversity of ECM fungi is high (210 species). About half of these fungi are generalists (117 species, 56%) and associated with *Dryas octopetala* as well as with the four alpine *Salix* species (Table S3). A smaller portion of fungi (38, 18%) is exclusively associated with calciphilic *D. octopetala* or with acidophilic *Salix herbacea* (23, 11%). A smaller portion is only associated with the four *Salix* species (32, 15%). Dominant genera are *Inocybe* with 99 species (49%) and *Cortinarius* with 54 species (26%). Besides the “SwissFungi” database, which included 130 species, the compilation of the French Alps from Kühner and Lamoure (1986) contributed considerably with 116 species to this list of ECM fungi.

### High-throughput DNA sequencing of alpine soils from Switzerland

The forefield of the “Damma” glacier (Rime et al., 2015) has several ECM forming plant species including *Salix herbacea* and *S. helvetica*, but also *Alnus viridis* and *Pinus mugo* at the lower margin of the site. Investigation of the soils resulted in 2,390 fungal operational taxonomic units (OTUs; a proxy to fungal species) in total. More than two thirds of the OTUs were assigned to the Ascomycetes, about 25% to the Basidiomycetes, and 5% remained unclassified. In the Basidiomycetes, a large group were Agaricales, with 120 OTUs, whereas Russulales, Boletales, and Cantharellales were less abundant. The majority of the classified Agaricales found were saprotrophic and not typical alpine species (e.g., *Marasmius rotula, Galerina marginata*). The genera *Cortinarius, Inocybe*, and *Entoloma*, with 5 to 6 OTUs each, were not particularly abundant, and some of their identified members were not typically known as alpine species (*Cortinarius decipiens, Inocybe lacera, I. jacobi, I. ochroalba, I. soluta, Entoloma griseocyaneum, E. pleopodium*, and *E. vernum*).

Frey et al. (2016), investigating the area near the summit of “Muot da Barba Peider,” observed only a few plant species of the genera *Poa, Cerastium* and *Senecio*. Thus, no ECM plants were present. In total, the authors recorded in the soils 840 fungal OTUs. However, about 20% could not be classified. About 400 of the classified OTUs were assigned to Ascomycetes, and 222 OTUs were assigned to Basidiomycetes. Fifty-three OTUs were assigned to Agaricales, with *Cortinarius, Inocybe*, and *Entoloma* having together 19 OTUs. The species *Cortinarius malicorius, C. uraceus, C. traganus, C. cotoneus, C. dilutus, Inocybe cookie*, and *I. soluta*, however, have ECM partners that are not in the alpine zone. Similarly, the recorded members of the Boletales have trees as ECM partners, which are not found in alpine areas.

## Discussion

Favre (1955) indicated for a majority of the recorded macrofungi that they grow in carpets of *Dryas octopetala* or *Salix* spp. Either these fungi are saprotrophs and decompose plant litter, or they form ECM associations with these plants. In particular due to this symbiotrophic relationship with the plant roots, the fungal flora in the alpine environment is remarkably rich. Favre (1955) lists about 90 ECM fungi in association with these hosts. Based on the fungal list of the Swiss, French, and German Alps including Favre (1955) and the “SwissFungi” database, we come to a total of 210 ECM taxa. With the 99 species of *Inocybe* and 54 species of *Cortinarius*, it cannot be excluded that some species are identical, in particular in *Cortinarius*, where microscopic characteristics are rare. In addition, the harsh alpine climatic conditions with strong rains, drought, snow, freezing and thawing, and high solar irradiation potentially strongly affect the morphological appearance of fruiting bodies. Therefore, colors, veil remnants, odor, and many other features might not be stable, which can result in vague descriptions and make identification of fungal species difficult, as already indicated by others (e.g., Horak, 1987b).

We are aware that there is more literature from the Alps, in particular from Italy. The book of Jamoni (2008) compiles a large collection of the fungi (193 taxa) recorded in the Italian Alps. Similarly as in Favre (1955), Jamoni (2008) listed the species taxonomically, and he added, besides outstanding pictures, taxonomical descriptions and ecological data such as associated plants. However, precise information about the sampling sites (coordinates, geology, elevation a.s.l.) is not given. Another noteworthy publication is from a glacier forefield of the Austrian Alps. Here, Horak (1960) listed 34 species and included taxonomically as well as ecological data.

Due to this high diversity of alpine fungi, it seems that there is truly a need for genetic characterisation of the type specimens. Up to now, however, <10 type specimens of Favre (1955) have been genetical characterized. In particular, with the upcoming new sequencing techniques well-founded databases are needed. It is one of the aims of the present study to highlight that type specimens of Favre (1955) are available at the botanical garden in Geneva. As an example of combining DNA analysis from deposited fungal fruiting bodies and modern HTS technologies, Geml et al. (2012) were able to identify more OTUs at the species level from an arctic environment. From the 109 phylogroups in total, the authors were able to identify and assign 62 to known species or species complexes, allowing them to detect more lineages than with either fruiting bodies or soil HTS alone.

About half of the 210 fungal ECM species in our list of the Swiss, French, and German Alps are generalist fungi (56%) associated with *Dryas octopetala* as well as with the four *Salix* species, and only a minority is associated with only one host plant, with *D. octopetala* (38 taxa, 18%) or with *Salix herbacea* (23 taxa, 11%). Based on the DNA identification of ECM root tips from the three ECM plants *D. octopetala, Salix polaris* and *Bistorta vivipara* in Svalbard (Norway), Botnen et al. (2014) came to a similar conclusion. Their analysis of the shared and unique OTUs showed that the majority of them (138 OTUs) were shared among the three EMC plants, whereas only between 24 and 38 OTUs were uniquely associated with each plant species, respectively.

Notwithstanding the extraordinary pioneering achievement of Favre (1955), his work has its weak points. Because he focussed on fruiting bodies easily visible to the naked eye, he clearly favored Basidiomycetes over Ascomycetes. In addition, his main sampling period was late summer, thus, the fruiting bodies of the spring and early summer period are lacking from his observation. Here, isolating fungal cultures or fungal DNA from soils or ECM root tips could potentially overcome this weakness (Brunner et al., 2011; Geml et al., 2012). For example, Geml et al. (2012) commonly encountered in arctic soils members of the order Sebacinales, which are missing in Favre's book, because their fruiting bodies were probably overlooked because they are very small or inconspicuous.

The potential of HTS techniques to record alpine fungi is given. Rime et al. (2015) in the glacier forefield recorded fungal taxa that are known from alpine and arctic regions, such as *Cortinarius aureomarginatus* (= *C chrysomallus*, = *C. saniosus), C. croceus*, and *C. diasemospermus* (Senn-Irlet, 1987; Graf, 1994; Lindström et al., 2008; Niskanen et al., 2008). Some other fungi also known from alpine regions were detected as well, e.g., *Psilocybe montana, Arrhenia lobata, A. griseopallida, Hygrocybe conica, H. miniata*, and *Galerina vittiformis*. However, in another HTS study with soils from the Swiss Alps (Frey et al., 2016), identification on a species level was rare.

New HTS techniques potentially can detect entire fungal communities of soils, and several bioinformatics tools and workflows provide quality filtering, clustering and identification, as well as sequence count matrices of OTUs (e.g., Schloss et al., 2009; Abarenkov et al., 2010). Nevertheless, problems associated with these new techniques are manifold, including sequencing errors, chimeric sequence formation during the polymerase chain reaction (PCR), and preferential amplification of taxa due to primer bias (see also Balint et al., 2016). An OTU is an operational definition used to classify groups of closely related individuals by similarity. At the genus level these identifications and comparisons are reliable, but at the species level the situation is often less clear. Ecological studies focused on individual species are usually based on an OTU similarity threshold ≥97% (see also Bjorbækmo et al., 2010). However, for species of the genera *Cortinarius* and *Inocybe*, it has been shown that similarity threshold ≥99% would yield more accurate results (Barge et al., 2016; Garnica et al., 2016).

Overall, we are convinced that there is an urgent need to improve databases with sequences from historic type material. As stated by Nilsson et al. (2016), precise and robust taxonomic assignment of many ITS sequences is not possible at present due to the lack of similar reference sequences in public databases, e.g., NCBI (National Center for Biotechnology Information; www.ncbi.nlm.nih.gov), INSDC (International Nucleotide Sequence Databases Collaboration; www.insdc.org), or UNITE (User-friendly Nordic ITS Ectomycorrhiza Database; unite.ut.ee). Thus, we propose an enhanced effort to sequence type material from fungal collections according to accepted standards, which include reliable sequence data combined with a correct taxonomic name as well as collection and voucher information (Schoch et al., 2014).

## Conclusion

The book of Favre (1955) represents a unique historical dataset of alpine macrofungal communities, and the combination with more recent fruiting body data provides a comprehensive compilation of ECM macrofungi of the Swiss, French and German Alps. In addition, we also identify the potential plant partners, with the conclusion, that many of the ECM taxa form symbiotrophic associations with more than one host plant. This fungal list can also potentially serve as a baseline for future alpine fungal ecological studies. Once the sequences of Favre's type specimens are incorporated in public DNA databases, they will enable a reliable identification of fungi in alpine soils analyzed with new HTS techniques. Thus, it is important to bridge historical data with new molecular methods for determining biodiversity of fungi in a region. Moreover, it will make it possible to assess possible shifts of alpine fungal communities under a changing climate when soil samples are taken at different time points in a standardized way and analyzed with standard HTS technologies. Such shifts have already been reported for alpine plant communities (e.g., Pauli et al., 2012; Wipf et al., 2013). It is likely that changing alpine plant communities will force the accompanying fungal communities to go along.

## Author contributions

IB, FG, and BS analyzed the data of Favre (1955). FG and BS contributed relevant fruiting body data of the Alps. BF and MH contributed relevant HTS data. SZ contributed relevant geology data of the SNP. LS, TN, and MB contributed signicifant information on OTUs recorded in alpine studies. All authors contributed to the writing.

### Conflict of interest statement

The authors declare that the research was conducted in the absence of any commercial or financial relationships that could be construed as a potential conflict of interest.
