# Supplementary material for: Ecology of Alpine Macrofungi - Combining Historical with Recent Data
Source: Front Microbiol. 2017 Oct 26;8:2066. doi: 10.3389/fmicb.2017.02066 (PMC5662630; doi:10.3389/fmicb.2017.02066)
Supplement: Supplementary file 1 [file Table1.PDF]

Supplementary Tables

Ecology of alpine macrofungi - combining historical with recent data

Ivano Brunner*, **Beat Frey, Martin Hartmann, Stephan Zimmermann, Frank Graf, Laura M. Suz, Tuula Niskanen, Martin I. Bidartondo and Beatrice Senn-Irlet**

***Correspondence:** Ivano Brunner: ivano.brunner@wsl.ch

Included in this file:

- **Table S1.** Number of records of rare ectomycorrhizal fungal species of Favre (1955).
- **Table S2.** Number of records of rare saprotrophic fungal species of Favre (1955).
- **Table S3.** Occurrence of ectomycorrhizal fungal taxa of the Swiss, French, and German Alps.

Table S1. Number of records of rare ectomycorrhizal fungal species (≤5 records) of Favre (1955). The macrofungi were growing in association with the calciphilic *Dryas octopetala (Dryas o.)* and *Salix reticulata, S. retusa* and *S. serpyllifolia (Salix rrs.*) on calcareous bedrock, and with the acidophilic *Salix herbacea* (*Salix h.*) on gneiss or verrucano. * Named by J. Favre.

Species name	Species name (in Favre 1955)	Fungal records in association with		
		Dryas o.	*Salix rrs.*	*Salix h.*
**In association with *Dryas octopetala* and all *Salix* species**				
Cortinarius cavipes *		1	1	3
Inocybe egenula *		1	1	1
Inocybe taxocystis *	*(=I. decipientoides var. taxocystis)*	2	1	2
Cortinarius favrexilis *	*(=C. glandicolor var. exilis)*	2	-	2
**In association with *Dryas octopetala* and calciphilic *Salix* species**				
Helvella ephippium		2	1	-
Inocybe flocculosa	*(=I. flocculosa, I. pallidipes)*	1	1	-
Inocybe rupestris *		1	1	-
Inocybe tenerella *	*(=I. ovalispora-subbrunnea f. tenerella)*	1	3	-
**In association with *Dryas octopetala***				
Cortinarius levipileus *		1	-	-
Cortinarius percavus *		1	-	-
Cortinarius phaeochrous *		1	-	-
Geopora foliacea	*(=Sepultaria foliacea)*	1	-	-
Geopora sumneriana	*(=Sepultaria lanuginosa)*	5	-	-
Hebeloma laterinum	*(=H. edurum)*	1	-	-
Helvella acetabulum	*(=Acetabula Barlae)*	4	-	-
Inocybe bongardii		1	-	-
Inocybe cervicolor		3	-	-
Inocybe decipientoides		1	-	-
Inocybe frigidula *		2	-	-
Inocybe leptocystis	*(=I. leptocystis var ambigua)*	2	-	-
Inocybe leucoblema		3	-	-
Inocybe lucifuga	*(=I. lucifuga var. lutescens)*	2	-	-
Inocybe luteipes *		1	-	-
Inocybe maculipes *		2	-	-
Inocybe monochroa *		1	-	-
Inocybe obscurobadia *	*(=I. furfurea var. obscurobadia)*	1	-	-
Inocybe ochroleuca *		1	-	-
Inocybe piricystis *		2	-	-
Inocybe pruinosa		2	-	-
Inocybe pseudohiulca		2	-	-
Inocybe rufobrunnea *		1	-	-
Inocybe rufolutea *		1	-	-
Russula foetens		2	-	-
Tricholoma argyraceum		1	-	-
Tricholoma terreum		1	-	-

Table S1. (continued)

Species name	Species name (in Favre 1955)	Fungal records in association with		
		Dryas o.	*Salix rrs.*	*Salix h.*
**In association with all *Salix* species**				
Cortinarius comatus *		-	3	1
Cortinarius rusticellus *		-	1	2
Cortinarius scotoides *		-	3	1
Inocybe concinnula *		-	1	1
**In association with calciphilic *Salix* species**				
Cortinarius gausapatus *		-	3	-
Cortinarius helvelloides		-	2	-
Cortinarius inops *		-	1	-
Helvella crispa		-	1	-
Inocybe geophylla		-	2	-
Russula graveolens	*(=R. xerampelina var. graveolens)*	-	1	-
**In association with *Salix herbacea***				
Cortinarius albonigrellus *		-	-	1
Cortinarius hemitrichus	*(=C. hemitrichus f. improcerus)*	-	-	1
Cortinarius inconspicuus *		-	-	1
Cortinarius oreobius *		-	-	2
Cortinarius pertristis *		-	-	1
Cortinarius rufostriatus *		-	-	1
Cortinarius subtilior *		-	-	1
Inocybe calamistrata		-	-	2
Inocybe favrei-cavipes *	*(=I. cavipes)*	-	-	1
Inocybe giacomi *		-	-	3
Inocybe napipes		-	-	1
Inocybe rufofusca *	*(=I. praetervisa f. rufofusca)*	-	-	2
Laccaria proxima		-	-	3
Lactarius violascens		-	-	1
Leptopodia capucina	*(=Helvella capucina)*	-	-	1
Naucoria tantilla *		-	-	1
Russula brunneoviolacea		-	-	1
Russula emetica	*(=R. emetica var. alpestris)*	-	-	2

Table S2. Number of records of rare saprotrophic fungal species (≤5 records) of Favre (1955). The macrofungi were growing in association with calciphilic *Dryas octopetala (D)* and/or *Salix reticulata, S. retusa* and *S. serpyllifolia (Srrs*), and/or with the acidophilic *Salix herbacea* (*Sh*), and/or in alpine grassland on calcareous (Gc) and/or acidic soils (Ga) and/or in bogs (B).
* Named by J. Favre.

Species name	Species name (in Favre 1955)	Fungal records in association with					
		D	*Srrs*	*Sh*	Gc	Ga	B
**Growing ubiquitous**							
Cystoderma amianthinum		1	1	1	1	1	-
Conocybe ochracea	*(=C. ochracea f. alpina)*	1	2	-	-	1	-
Entoloma catalaunicum	*(=Rhodophyllus catalaunicus)*	1	2	-	1	-	-
Entoloma chalybeum	*(=Rhodophyllus chalybaeus)*	1	1	-	-	1	-
Entoloma griseocyaneum	*(=Rhodophyllus griseocyaneus)*	1	-	-	-	1	-
Entoloma incanum	*(=Rhodophyllus incanus)*	3	-	-	1	-	-
Entoloma serrulatum	*(=Rhodophyllus serrulatus)*	1	-	-	-	1	-
Entoloma turci	(*=Rhodophyllus Turci*)	2	-	-	1	-	-
Clitocybe infundibuliformis		2	-	-	1	2	-
Hygrocybe miniata	*(=Hygrophorus miniatus)*	1	-	-	3	-	-
Mycena pura		1	-	-	-	1	-
Arrhenia griseopallida	*(=Omphalina griseopallida)*	-	1	-	-	1	-
Hygrocybe aurantiosplendens	*(=Hygrophorus aurantiosplendens)*	-	1	-	1	1	-
Entoloma anthracinum *	*(=Rhodophyllus anthracinus)*	-	-	1	1	-	-
Alboleptonia sericella	*(=Rhodophyllus sericellus)*	-	-	1	1	-	-
Galerina vittiformis	*(=Galera rubiginosa f. muricellospora)*	-	-	1	1	-	-
Hygrocybe flavescens	*(=Hygrophorous flavescens)*	-	-	1	-	1	-
**Growing with *Dryas octopetala* and all *Salix* species**							
Clavulina cinerea	*(=Clavaria cinerea)*	1	-	-	-	-	-
Clitocybe festiva *		4	-	-	-	-	-
Geastrum minimum		5	-	-	-	-	-
Gymnopus impudicus	*(=Marasmius impudicus)*	1	-	-	-	-	-
Heydenia alpina		3	-	-	-	-	-
Lepiota pseudolilacea	*(=Lepiota pseudohelveola)*	4	-	-	-	-	-
Marasmius amethystinus *		1	-	-	-	-	-
Mycenella salicina	*(=Mycena salicina)*	1	-	-	-	-	-
Ripartites tricholoma		1	-	-	-	-	-
Tulostoma brumale		1	-	-	-	-	-
Tulostoma squamosum		1	-	-	-	-	-
Entoloma clandestinum	*(=Rhodophyllus clandestinus)*	1	-	-	-	-	-
Entoloma poliopus	*(=Rhodophyllus poliopus f. alpigenus)*	2	1	-	-	-	-
Conocybe aberrans		-	1	-	-	-	-
Entoloma vernum	*(=Rhodophyllus cucullatus)*	-	1	-	-	-	-
Entoloma atropellitus *	*(=Rhodophyllus atropellitus)*	-	-	1	-	-	-
**Growing in all grasslands**							
Agaricus campestris	*(=Psalliota campestris)*	-	-	-	1	1	-
Calocybe gambosa	*(=Lyophyllum Georgii)*	-	-	-	1	1	-
Calvatia utriformis	*(=C. coelata)*	-	-	-	2	1	-
Cuphophyllus virgineus	*(=Hygrophorus virgineus)*	-	-	-	1	3	-
Lepista irina	*(=Rhodopaxillus irinus)*	-	-	-	1	1	-
Melanoleuca grammopodia		-	-	-	1	1	-
Psilocybe inquilina	*(=Geophila inquilina)*	-	-	-	1	1	-

Table S2. (continued)

Species name	Species name (in Favre 1955)	Fungal records in association with					
		D	*Srrs*	*Sh*	Gc	Ga	B
**Growing in grassland on calcareous soils**							
Arrhenia auriscalpium		-	-	-	1	-	-
Conocybe tenera		-	-	-	1	-	-
Entoloma whiteae	*(=Rhodophyllus Whiteae)*	-	-	-	1	-	-
Deconica velifera *	*(=Geophila velifera)*	-	-	-	1	-	-
Helvella lacunosa		-	-	-	1	-	-
Lepiota erminea	*(=L. alba)*	-	-	-	2	-	-
Lepista panaeolus	*(=Rhodopaxillus panaeolus)*	-	-	-	2	-	-
Lycoperdon lividum	*(=L. spadiceum)*	-	-	-	1	-	-
Melanoleuca melaleuca		-	-	-	5	-	-
Protostropharia semiglobata	*(=Geophila semiglobata)*	-	-	-	2	-	-
**Growing in grassland on acidic soils**							
Arrhenia latispora *	*(=Pleurotellus acerosus f. latisporus)*	-	-	-	-	1	-
Clitocybe bresadolana	*(=C. Bresadoliana)*	-	-	-	-	1	-
Clitocybe metachroa	*(=C. aff. mortuosa)*	-	-	-	-	1	-
Galerina hypnorum	*(=Galera hypnorum)*	-	-	-	-	1	-
Gliophorus laetus	*(=Hygrophorus laetus)*	-	-	-	-	2	-
Gliophorus psittacinus	*(=Hygrophorus psittacinus)*	-	-	-	-	2	-
Gymnopus fuscopurpureus	*(=Marasmius aff. fuscopurpureus)*	-	-	-	-	1	-
Hemimycena ochrogaleata *	*(=Mycena ochrogaleata)*	-	-	-	-	2	-
Hygrocybe coccinea	*(=Hygrophorus coccineus)*	-	-	-	-	2	-
Hygrocybe nitrata	*(=Hygrophorus nitratus)*	-	-	-	-	1	-
Hygrocybe punicea	*(=Hygrophorus puniceus)*	-	-	-	-	1	-
Lycoperdon perlatum	*(=L. gemmatum)*	-	-	-	-	1	-
Omphalina brownii		-	-	-	-	1	-
Omphalina pyxidata		-	-	-	-	1	-
Omphalina rivulicola *	*(=Omphalina pyxidata var. rivulicola)*	-	-	-	-	2	-
Panaeolina foenisecii	*(=Panaeolus foenisecii)*	-	-	-	-	2	-
Rhodocollybia butyracea	*(=Collybia butyracea)*	-	-	-	-	1	-
**Growing in bogs**							
Arrhenia acerosa	*(=Pleurotellus acerosus)*	-	-	-	-	-	1
Arrhenia umbratilis	*(=Omphalina umbratilis)*	-	-	-	-	-	3
Bovistella paludosa		-	-	-	-	-	2
Bryoglossum gracile	*(=Mitrula gracilis)*	-	-	-	-	-	4
Coprinus Martinii *		-	-	-	-	-	1
Galerina pumila	*(=Galera pumila f. oreina)*	-	-	-	-	-	2
Humaria hemisphaerica	*(=Lachnea hemisphaerica)*	-	-	-	-	-	1
Phaeogalera stagnina	*(=Galera stagnina var. pallida)*	-	-	-	-	-	1
Psilocybe elongata	*(=Geophila elongata)*	-	-	-	-	-	4
**Preferentially growing in bogs**							
Panaeolus acuminatus		-	-	-	-	1	2
Arrhenia lobata	*(=Leptoglossum lobatum)*	-	1	-	-	-	2
Galerina clavata	*(=Galera clavata)*	-	-	1	-	-	2
Rickenella fibula	*(=Omphalina fibula)*	-	-	1	-	-	3

Table S3. Occurrence of ectomycorrhizal fungal taxa of the Swiss, French, and German Alps. The macrofungi were growing in association with the calciphilic *Dryas octopetala (D)* and *Salix reticulata, S. retusa* and *S. serpyllifolia (Srrs*), and with the acidophilic *Salix herbacea* (*Sh*). The sources of information are: SwissFungi16='SwissFungi' Database 2016, Favre55=Favre (1955), KL86=Kühner and Lamoure (1986), SH85=Schmid-Heckel (1985), GR94=Graf (1994), SI87=Senn-Irlet (1987), BG73=Bon and Géhu (1973). (x?: the association is not specifically indicated).

Species name	SwissFungi16			Favre55			KL86			SH85		GR94	SI87	BG73
	D	*Srrs*	*Sh*	*D*	*Srrs*	*Sh*	*D*	*Srrs*	*Sh*	*D*	*Srrs*	*Sh*	*Sh*	*Sh*
**In association with *Dryas octopetala***														
Cortinarius delibutus	x	-	-	-	-	-	x	-	-	-	-	-	-	-
Cortinarius glandicolor	x	-	-	-	-	-	-	-	-	-	-	-	-	-
Cortinarius infractus	-	-	-	-	-	-	x	-	-	-	-	-	-	-
Cortinarius levipileus	x	-	-	x	-	-	x	-	-	-	-	-	-	-
Cortinarius polaris	x	-	-	-	-	-	-	-	-	-	-	-	-	-
Cortinarius traganus	x	-	-	-	-	-	-	-	-	-	-	-	-	-
Cortinarius violaceorubens	x	-	-	-	-	-	-	-	-	-	-	-	-	-
Cortinarius violeovelatus	-	-	-	-	-	-	x	-	-	-	-	-	-	-
Hebeloma crustuliniforme	x	-	-	-	-	-	-	-	-	-	-	-	-	-
Hebeloma laterinum	x	-	-	-	-	-	-	-	-	-	-	-	-	-
Helvella acetabulum	-	-	-	x	-	-	-	-	-	-	-	-	-	-
Inocybe abjecta	x	-	-	-	-	-	-	-	-	-	-	-	-	-
Inocybe acuta	-	-	-	-	-	-	x	-	-	-	-	-	-	-
Inocybe albofibrillosa	-	-	-	-	-	-	-	-	-	x	-	-	-	-
Inocybe appendiculata	x	-	-	-	-	-	-	-	-	-	-	-	-	-
Inocybe bongardii	x	-	-	-	-	-	-	-	-	-	-	-	-	-
Inocybe erubescens (=I. patouillardii)	-	-	-	-	-	-	x	-	-	-	-	-	-	-
Inocybe flavella	x	-	-	-	-	-	-	-	-	-	-	-	-	-
Inocybe fraudans	x	-	-	-	-	-	-	-	-	-	-	-	-	-
Inocybe furfurea	-	-	-	-	-	-	x	-	-	-	-	-	-	-
Inocybe fuscidula	x	-	-	-	-	-	-	-	-	-	-	-	-	-
Inocybe griseolilacina	x	-	-	-	-	-	-	-	-	-	-	-	-	-
Inocybe leptocystis	x	-	-	-	-	-	-	-	-	-	-	-	-	-
Inocybe malenconii (=I. malençoni)	-	-	-	-	-	-	x	-	-	-	-	-	-	-
Inocybe maculipes	x	-	-	x	-	-	-	-	-	-	-	-	-	-
Inocybe oblectabilis	x	-	-	-	-	-	-	-	-	-	-	-	-	-
Inocybe olivaceobrunnea	x	-	-	-	-	-	-	-	-	-	-	-	-	-
Inocybe pelargonium	x	-	-	-	-	-	-	-	-	-	-	-	-	-
Inocybe substraminipes	x	-	-	-	-	-	-	-	-	-	-	-	-	-
Inocybe terrigena	x	-	-	-	-	-	-	-	-	-	-	-	-	-
Inocybe vulpinella	x	-	-	-	-	-	-	-	-	-	-	-	-	-
Lactarius deterrimus	-	-	-	-	-	-	-	-	-	x	-	-	-	-
Lactarius subcircellatus	x	-	-	-	-	-	-	-	-	-	-	-	-	-
Lactarius trivialis	x	-	-	-	-	-	-	-	-	-	-	-	-	-
Russula chloroides	x	-	-	-	-	-	-	-	-	-	-	-	-	-
Russula cuprea	x	-	-	-	-	-	-	-	-	-	-	-	-	-
Russula pallidospora	x	-	-	-	-	-	-	-	-	-	-	-	-	-
Tricholoma argyraceum	x	-	-	x	-	-	x	-	-	-	-	-	-	-

Table S3. (continued)

Species name	SwissFungi16			Favre55			KL86			SH85		GR94	SI87	BG73
	D	*Srrs*	*Sh*	*D*	*Srrs*	*Sh*	*D*	*Srrs*	*Sh*	*D*	*Srrs*	*Sh*	*Sh*	*Sh*
**In association with *Salix herbacea***														
Cortinarius bresadolae	-	-	-	-	-	-	-	-	x	-	-	-	-	-
Cortinarius caesionigrellus	-	-	-	-	-	-	-	-	x	-	-	x	-	-
Cortinarius cyanites	-	-	-	-	-	-	-	-	x	-	-	-	-	-
Cortinarius diasemospermus	-	-	-	-	-	-	-	-	x	-	-	x	-	-
Cortinarius hemitrichus	-	-	-	-	-	-	-	-	x	-	-	-	-	-
Cortinarius lamoureae (=C. fallax)	-	-	-	-	-	-	-	-	x	-	-	x	-	-
Cortinarius pertristis	-	-	-	-	-	x	-	-	-	-	-	x	-	x
Cortinarius rufostriatus	-	-	-	-	-	x	-	-	-	-	-	x	x	-
Hebeloma nigellum	-	-	-	-	-	-	-	-	x	-	-	-	-	-
Hymenogaster saliciphilus	-	-	x	-	-	-	-	-	-	-	-	x	-	-
Inocybe curvipes	-	-	-	-	-	-	-	-	-	-	-	x	-	-
Inocybe hygrophana	-	-	x	-	-	-	-	-	-	-	-	-	-	-
Inocybe ovatocystis	-	-	-	-	-	-	-	-	-	-	-	x	-	-
Inocybe rufofusca	-	-	x	-	-	-	-	-	-	-	-	-	-	-
Inocybe striaepes	-	-	-	-	-	-	-	-	x	-	-	-	-	-
Inocybe substellata	-	-	x	-	-	-	-	-	-	-	-	-	-	-
Laccaria bicolor	-	-	-	-	-	-	-	-	-	-	-	x	-	-
Laccaria montana	-	-	-	-	-	x	-	-	-	-	-	x	x	-
Laccaria proxima	-	-	-	-	-	x	-	-	-	-	-	x	-	-
Lactarius pseudouvidus	-	-	-	-	-	-	-	-	x	-	-	-	-	-
Naucoria bohemica	-	-	-	-	-	-	-	-	-	-	-	x	-	-
Russula nauseosa	-	-	-	-	-	-	-	-	x	-	-	-	-	-
Thelephora terrestris	-	-	-	-	-	-	-	-	-	-	-	x	-	-
**In association with all *Salix* species**														
Amanita oreina	-	-	-	-	x	x	-	-	-	-	-	x	-	x
Cortinarius albonigrellus	-	-	-	-	-	x	-	x	x	-	-	-	-	x
Cortinarius fulvescens	-	-	-	-	-	-	-	x	-	-	-	-	-	-
Cortinarius galerinoides	-	-	-	-	-	-	-	x	x	-	-	-	-	-
Cortinarius helvelloides	-		-	-	x	-	-	-	-	-	-	-	-	-
Cortinarius purpureoluteus	-	-	-	-	-	-	-	x	x	-	-	-	-	-
Heb. bruchetii (=H. repandum)	-	-	-	-	-	-	-	x	x	-	-	-	-	-
Hebeloma kuehneri	-	-	-	-	-	-	-	x	x	-	-	-	-	-
Hebeloma subconcolor	-	-	-	-	-	-	-	x	-	-	-	-	-	-
Helvella crispa	-	-	-	-	x	-	-	-	-	-	-	-	-	-
Inocybe alboperonata	-	x	-	-	-	-	-	-	-	-	-	-	-	-
Inocybe auricomella	-	-	x	-	-	-	-	x	x	-	-	-	-	-
Inoc. cincinnata (=I. cincinnatula)	-	-	-	-	-	-	-	x	-	-	-	-	-	-
Inocybe dulcamaroides	-	-	-	-	-	-	-	x	x	-	-	-	-	-
Inocybe godfrinioides	-	-	-	-	-	-	-	x	x	-	-	-	-	-
Inocybe guttulifer	-	x	x	-	-	-	-	x	-	-	-	-	-	-
Inocybe iseranensis	-	x	-	-	-	-	-	-	-	-	-	-	-	-
Inocybe jacobi	-	x	-	-	-	-	-	-	-	-	-	-	-	-
Inocybe lutescens	-	x	-	-	-	-	-	x	-	-	-	-	-	-
Inocybe moelleri	-	x	-	-	-	-	-	-	-	-	-	-	-	-
Inocybe paludosa	-	-	x	-	-	-	-	x	-	-	-	-	-	-
Inocybe pelargoniodora	-	-	-	-	-	-	-	x	-	-	-	-	-	-
Inocybe rufolutea	-	x	-	-	-	-	-	-	-	-	-	-	-	-
Inocybe rupestris	-	-	-	-	x	-	-	-	-	-	-	-	-	-
Inocybe subannulata	-	-	-	-	-	-	-	x	-	-	-	-	-	-
Inocybe subbrunnea	-	-	x	-	-	-	-	x	x	-	-	-	-	-
Inocybe subfusca	-	-	-	-	-	-	-	x	-	-	-	-	-	-
Inocybe tetragonospora	-	x	-	-	-	-	-	-	-	-	-	-	-	-
Inocybe umbrinofusca	-	x	-	-	-	-	-	x	x	-	-	-	-	-
Lactarius uvidus	-	-	-	-	-	-	-	x	x	-	-	-	x	x
Russula graveolens	-	-	-	-	x	-	-	-	-	-	-	-	-	-
Russula laccata (=R. norvegica)	-	-	-	-	-	-	-	x	x	-	-	x	x	-

Table S3. (continued)

Species name	SwissFungi16			Favre55			KL86			SH85		GR94	SI87	BG73
	D	*Srrs*	*Sh*	*D*	*Srrs*	*Sh*	*D*	*Srrs*	*Sh*	*D*	*Srrs*	*Sh*	*Sh*	*Sh*
**In association with *Dryas octopetala* and all *Salix* species**														
Amanita nivalis	x	x	x	-	-	-	-	-	x	-	-	-	-	-
Amanita vaginata (=A. hyperborea)	x	x	x	-	-	-	-	-	x	-	x	-	-	-
Cortinarius alpicola	x	x	-	-	-	-	-	-	-	-	-	-	-	-
Cortinarius alpinus (=C. favrei)	x	x	-	-	x	-	x	x	x	x	x	x	x	x
Cortinarius anomalus	x	x	-	x	x	x	x	x	x	x	-	x	-	-
Cortinarius calcialpinus	x	x	-	-	-	-	-	-	-	-	-	-	-	-
Cortinarius cavipes	-	-	-	x	x	x	x?	x?	x?	-	-	x	-	-
Cortinarius chamaesalicis	x	-	-	-	-	-	-	x	x	-	-	-	-	-
Cort. chrysomallus (=C. saniosus)	-	-	-	-	-	-	x	-	x	-	-	x	x	-
Cortinarius cinnamomeoluteus	-	-	-	-	-	x	x	x	x	-	-	x	x	-
Cortinarius cinnamomeus	x	-	-	-	x	x	-	-	-	-	-	-	-	-
Cortinarius comatus	x	x	-	-	x	-	-	x	-	-	-	-	-	-
Cortinarius croceus	-	-	-	-	-	-	-	-	-	x	-	x	-	-
Cortinarius favrexilis	x	-	-	x	-	x	-	-	-	-	-	x	-	-
Cortinarius flexipes (=C. paleiferus)	x	-	-	-	-	-	-	-	x	-	-	-	-	-
Cortinarius gausapatus	x	x	-	-	x	-	-	x	x	-	-	x	-	-
Cortinarius hinnuleus	x	x	x	x	x	x	x	x	x	-	-	x	x	-
Cortinarius inconspicuus	x	-	-	-	-	x	-	-	-	-	-	-	-	-
Cortinarius inops	-	-	-	-	x	-	x	x	x	-	-	-	-	-
Cortinarius minutalis	-	-	-	-	-	-	x	x	x	x	-	-	-	-
Cortinarius minutulus	x	x	x	x	x	x	x	-	-	-	-	x	-	-
Cortinarius oreobius	x	-	-	-	-	x	-	-	-	-	-	-	-	x
Cort. parvannulatus (=C. cedriolens)	-	-	-	-	-	-	x	x	-	-	-	-	-	-
Cortinarius pauperculus	x	-	-	x	x	x	x	x	x	-	-	x	x	-
Cortinarius percavus	x	-	x	x	-	-	-	-	-	-	-	x	x	-
Cortinarius phaeochrous	x	x	-	x	-	-	x	-	-	-	-	-	-	-
Cortinarius phaeopygmaeus	x	x	-	-	x	x	-	-	-	-	-	x	-	-
Cortinarius pulchripes	-	-	-	-	-	-	x	x	x	-	-	-	-	-
Cortinarius pusillus	x	x	x	-	-	-	-	-	-	-	-	-	-	-
Cortinarius rusticellus	x	-	-	-	x	x	x	x	x	-	-	x	-	-
Cortinarius scotoides	x	x	-	-	x	-	-	x	-	-	-	-	-	-
Cortinarius stenospermus	-	-	-	-	-	-	x	x	-	-	-	-	-	-
Cortinarius subtilior	-	-	-	-	-	x	x	x	x	-	-	-	-	x
Cortinarius subtorvus	x	x	-	-	-	-	x	x	-	-	-	-	-	-
Cortinarius tenebricus	x	x	-	x	x	x	-	x	x	-	x	x	-	-
Hebeloma alpinum	x	x	-	x	x	x	x	x	x	x	-	-	-	-
Hebeloma marginatulum	x	x	-	x	x	x	-	x	x	-	-	x	x	-
Hebeloma mesophaeum	x	x	-	-	x	-	-	-	-	-	-	-	-	-
Hebeloma minus	x	-	-	-	-	-	-	x	x	-	-	-	x	-
Hebeloma remyi	x	-	x	-	-	-	x?	x?	x?	-	-	-	-	-
Helvella corium	x	x	x	x	x	x	-	-		-	-	-	-	-
Helvella ephippium	-	-	-	x	x	-	-	-	-	-	-	-	-	-
Inocybe alpigenes	x	x	-	-	-	-	-	-	-	-	-	-	-	-
Inocybe amoenolens	-	-	-	-	-	-	x	x	-	-	-	-	-	-
Inocybe arthrocystis	-	-	-	-	-	-	x	x	-	-	-	-	-	-
Inocybe bivela	-	-	-	-	-	-	x	x	-	-	-	-	-	-
Inocybe calamistrata	x	-	x	x	-	-	x	x	x	x	-	x	x	-
Inocybe canescens	-	-	x	-	x	-	x	x	x	-	x	-	-	-
Inocybe catalaunica	x	x	-	-	-	-	-	-	-	-	-	-	-	-
Inocybe concinnula	x	x	x	-	x	x	x	x	-	-	-	-	-	-
Inocybe decipiens	x	-	-	-	x	-	-	-	-	x	x	-	-	-
Inocybe dulcamara	x	x	-	x	x	x	x	x	-	-	-	-	-	x
Inocybe egenula	x	x	-	x	x	x	-	-	-	-	-	x	-	-
Inocybe favrei	x	x	x	-	-	-	-	-	-	-	-	-	-	-
Inocybe flocculosa	-	-	-	-	x	-	x	x	-	-	-	-	-	-
Inocybe frigidula	x	x	-	x	-	-	-	-	-	x	-	-	-	-
Inocybe fulvipes	x	x	-	-	-	-	-	-	-	-	-	-	-	-
Inocybe fuscescentipes	x	-	x	-	-	-	-	-	-	-	-	-	-	-
Inocybe fuscomarginata	x	-	-	-	x	-	x	x	x	-	-	-	-	-
Inocybe geophylla	-	-	-	-	x	-	x	x	x	-	-	-	-	-
Inocybe geraniodora	x	x	-	x	x	x	-	x	-	x	-	-	-	x
Inocybe giacomi	x	x	x	-	-	x	-	-	x	-	-	x	x	x
Inocybe grata	-	-	-	-	-	-	x	x	-	-	-	-	-	-

Table S3. (continued)

Species name	SwissFungi16			Favre55			KL86			SH85		GR94	SI87	BG73
	D	*Srrs*	*Sh*	*D*	*Srrs*	*Sh*	*D*	*Srrs*	*Sh*	*D*	*Srrs*	*Sh*	*Sh*	*Sh*
**In association with *Dryas octopetala* and all *Salix* species (continued)**														
Inocybe heterocystis	-	-	-	-	-	-	x	x	-	-	-	-	-	-
Inocybe immaculipes	-	-	-	-	-	-	x	x	-	-	-	-	-	-
Inocybe inodora	x	x	-	-	-	-	-	-	-	-	-	-	-	-
Inocybe johannae	x	-	-	-	-	-	-	x	-	-	-	-	-	-
Inocybe lacera (=I. rhacodes)	x	-	x	x	-	x	-	x	x	-	-	x	x	-
Inocybe leucoblema	x	x	-	-	-	-	x	-	-	-	-	-	-	-
Inocybe melliolens	-	-	-	-	-	-	x	x	-	-	-	-	-	-
Inocybe microfastigiata	x	x	-	-	-	-	x	x	x	-	-	-	-	-
Inocybe monochroa	x	x	-	x	-	-	-	-	-	-	-	-	-	-
Inocybe mundula	-	-	-	x	-	-	x	x	x	-	-	x	-	-
Inocybe napipes	x	x	-	-	-	-	-	-	-	-	-	-	-	-
Inocybe nitidiuscula (=I. friesii)	x	x	-	x	x	-	x	x	x	-	-	x	-	-
Inocybe oreina	x	-	-	x	x	-	x	x	-	-	-	-	-	-
Inocybe peronatella	-	-	-	-	-	-	x	-	-	-	-	x	-	-
Inocybe petiginosa	-	-	-	-	-	-	x	x	-	-	-	-	-	-
Inocybe piricystis	-	-	-	x	-	-	-	-	-	-	-	x	-	-
Inocybe posterula	x	x	-	-	-	-	-	-	-	-	-	-	-	-
Inocybe praetervisa	x	-	x	-	x	x	-	-	-	x	-	-	-	-
Inocybe pruinosa	-	-	-	x	-	-	-	-	-	-	-	x	-	-
Inocybe pseudohiulca	x	x	-	x	-	-	-	-	-	-	-	-	-	-
Inocybe rhacodes	x	-	-	-	-	x	-	-	-	-	-	-	-	-
Inocybe rimosa (=I. fastigiata)	x	x	-	x	x	x	x	x	x	x	-	x	-	-
Inocybe rufobrunnea	x	x	-	x	-	-	-	-	-	-	-	-	-	-
Inocybe salicis-herbaceae	-	x	-	-	-	-	x	-	x	-	-	-	-	-
Inocybe similis	x	x	-	-	-	-	-	-	-	-	-	-	-	-
Inocybe sindonia (=I. eutheles)	-	-	-	-	-	-	x	x	-	-	-	-	-	-
Inocybe squarrosoannulata	x	-	-	-	-	-	x	x	x	-	-	-	-	-
Inocybe submaculipes	-	-	-	-	-	-	x	x	-	-	-	-	-	-
Inocybe subpaleacea	-	-	-	-	-	-	x	x	-	-	-	-	-	-
Inocybe taxocystis	-	-	x	x	x	x	-	-	-	-	-	x	-	-
Inocybe tenerella	x	-	-	-	x	-	x	x	-	-	-	-	-	-
Inocybe tjallingiorum	x	x	-	-	-	-	-	-	-	-	-	-	-	-
Inocybe tricolor	-	-	-	-	-	-	x	-	x	-	-	-	-	-
Laccaria laccata	x	x	-	x	x	x	-	x	x	-	-	-	-	-
Laccaria pumila (=L. altaica)	x	x	-	-	x	-	x	x	-	-	x	-	-	-
Laccaria tetraspora	x	-	-	-	-	-	x?	x?	x?	-	-	-	x	-
Laccaria tortilis	-	-	-	-	-	-	x?	x?	x?	-	-	-	-	-
Lactarius brunneoviolaceus	x	x	-	-	-	-	-	-	-	-	-	-	-	-
Lactarius dryadophilus	x	x	-	-	-	-	x	x	-	x	x	-	-	-
Lactarius nanus	x	x	x	-	-	x	-	-	x	-	-	-	x	x
Lactarius salicis-herbaceae	x	-	-	-	-	-	-	-	x	-	-	-	-	-
Lactarius salicis-reticulatae	x	x	-	-	-	-	-	x	-	-	-	-	-	-
Naucoria tantilla (=N. chamiteae)	-	-	-	-	-	x	x	x	x	-	-	-	-	x
Russula delica	x	x	-	-	-	-	x	-	-	x	-	-	-	-
Russula dryadicola	x	x	x	-	-	-	-	-	-	-	-	-	-	-
Russula emetica	x	x	-	-	-	-	-	-	-	-	-	-	-	-
Russula maculata	x	-	-	-	-	-	x?	x?	x?	-	-	-	-	-
Russula nana	x	x	-	-	-	x	-	x	x	x	-	-	x	-
Russula pascua	x	x	-	-	-	x	-	-	x	-	-	x	-	-
Russula saliceticola	x	x	-	-	-	-	-	-	-	-	-	-	-	-
Russula subrubens (=R. chamitae)	x	x	-	-	-	-	-	-	x	-	-	x	-	-
Thelephora anthocephala	x	x	-	-	-	-	-	-	-	-	-	-	-	-
Thelephora caryophyllea	x	x	-	-	-	-	-	-	-	-	-	-	-	-
Tricholoma sulphureum	x	x	-	-	-	-	x	-	-	-	-	-	-	-
